# A Neuro-Fuzzy System for Extracting Environment Features Based on Ultrasonic Sensors

**DOI:** 10.3390/s91210023

**Published:** 2009-12-09

**Authors:** Graciliano Nicolás Marichal, Angela Hernández, Leopoldo Acosta, Evelio José González

**Affiliations:** Departamento de Ingeniería de Sistemas y Automática, Arquitectura y Tecnología de Computadoras, Universidad de La Laguna, La Laguna, Tenerife 38071, Spain; E-Mails: angela@isaatc.ull.es (A.H.); lacosta@ull.es (L.A.); evelio@isaatc.ull.es (E.G.)

**Keywords:** neural networks, fuzzy systems, neuro-fuzzy, ultrasonic sensing, autonomous robots

## Abstract

In this paper, a method to extract features of the environment based on ultrasonic sensors is presented. A 3D model of a set of sonar systems and a workplace has been developed. The target of this approach is to extract in a short time, while the vehicle is moving, features of the environment. Particularly, the approach shown in this paper has been focused on determining walls and corners, which are very common environment features. In order to prove the viability of the devised approach, a 3D simulated environment has been built. A Neuro-Fuzzy strategy has been used in order to extract environment features from this simulated model. Several trials have been carried out, obtaining satisfactory results in this context. After that, some experimental tests have been conducted using a real vehicle with a set of sonar systems. The obtained results reveal the satisfactory generalization properties of the approach in this case.

## 1. Introduction

Several environment recognition techniques have been developed by different authors [1,2], and their development requires sensors that are able to detect obstacles and to measure distances. Some examples of applications of these techniques are parking aids, automatic navigation, building of environment maps, *etc.* An interesting option is the use of ultrasonic sensors. Ultrasonic sensors have been widely used in collision avoidance systems, in addition to the localization and navigation of mobile robots. These sensors are less expensive than others and it makes them appropriate for general applications in vehicles and autonomous mobile robots [3-6]. The fundamental principle of ultrasonic sensors is the time of flight technique which consists of determining the distance to a reflective surface by emitting high-frequency sound waves and measuring the time it takes for the echo to be picked up by the detector. The advantage to use these sensors is that they are reasonably cheap, easy to interface to computers and work for ranges of a few centimeters to a few meters, which explains their widespread use. In fact, it is easy to obtain distance information from immediate objects without intensive processing. Furthermore, they are able to perform under low visibility conditions. Because of that, they have often been used in navigation and localization problems of mobile robots [5]. In this framework several approaches to build maps have been proposed. The approaches can be categorized into two groups: metric and topological approaches. In the first case, the approaches focused on extracting the geometrical features of the environment, whereas, in the second case, topological maps are built, in order to depict the connections between the different environment parts. In the first case, the construction of an occupancy grid where the environment is discretized in an array of cells is very usual. Many approaches based on extracting several features from the geometry of the environment have been proposed. In recent years, some approaches have emerged where an extension to the 3D space have been incorporated [7,8]. However, a drawback in these 3D approaches lies on the high computational charge when the occupancy grid strategy is applied. In this paper, an intelligent approach based on a 3D model of the environment is presented, where the emphasis is done in the extraction of features. In fact, the research has been focused on determining walls and corners. In the text the walls are considered as the extension of a line segment lying on a plane, whereas, the corners are considered as the intersection of two planes, being observed from inside the concave space. At a first stage of the work, a 3D model of a particular environment has been built. This 3D model has been used as data source for the learning phases of the Neuro-Fuzzy approach and a first platform to test the methods shown in the paper. Once, the simulation tests have provided satisfactory results, new experiments with a real vehicle have been carried out.

In Section 2, the simulated 3D model used in the subsequent sections is presented. Section 3 is focused on the presentation of the used Neuro-Fuzzy system, while Section 4 will focus on the application of the Neuro-Fuzzy system and presentation of the results obtained. Conclusions are outlined in Section 5.

## 2. Simulated Environment

**Figure 1.**
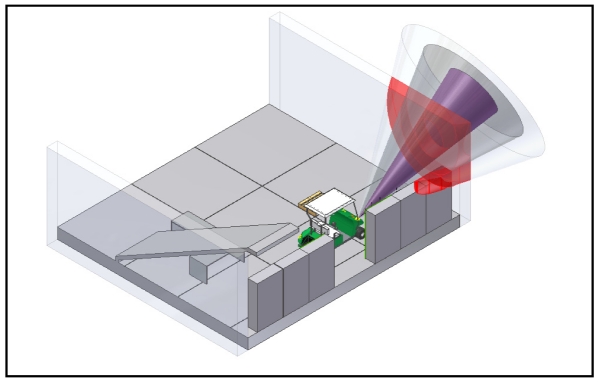
Image of the workstore.

**Figure 2.**
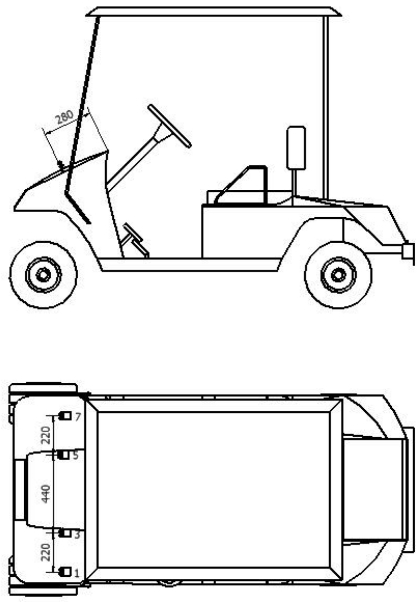
Image of the vehicle with the sonar system. The distances are given in millimeters.

Simulation of the environment is the first step in this work. In this case, the CAD software Autodesk Inventor has been chosen [9]. Graphical visualization is especially important when working in 3D. In order to test the techniques used in this paper, a workplace has been modeled. Furthermore, a vehicle along with their sonar systems on the bodywork has been incorporated into the 3D model. Moreover, objects inside the workplace such as cans, closets, *etc.* have also been modeled. A faithful carbon copy of the real environment has been done, overall taking into account all geometrical details. In Figure 1, an image of the simulated model is shown. An important aspect of the 3D model is the incorporation of the sonar systems. As it can be seen in Figure 2 several sonar systems has been included in the model. Only, sonar systems on the front part have been used in this work, although other sonar systems could be added in other parts of the vehicle. In fact, a faithful copy of the model TXT-2 shuttle manufactured by EZGO has been considered, given that this vehicle has been used in the field tests. Although, a particular environment has been modeled, it is important to remark that the final purpose of the shown approach is to determine common features of this environment in other ones. Because of that, the work has been focused on walls and corners as it was pointed out above. They are common features in many scenarios.

**Figure 3.**
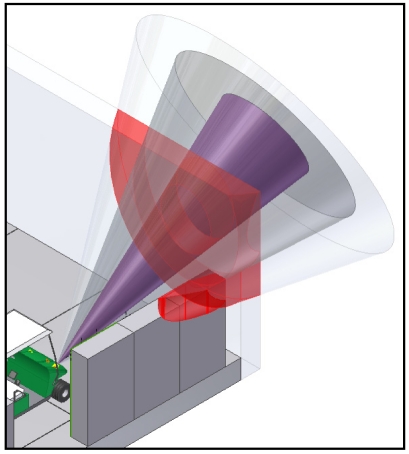
Image of the sonar cone and the different cone zones.

Particularly, SRF08 sonar systems have been chosen. As it is usual in most approaches in robotics, a basic model of a sonar cone has been assumed. In Figure 3, an image of the sonar cone is shown, where the different zones of the cone have been distinguished by different colors.

**Figure 4.**
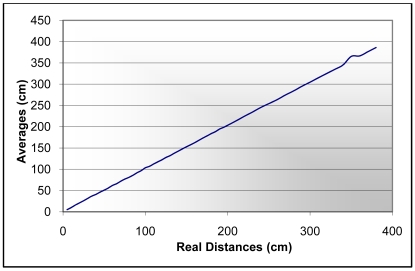
Averages of several samples *vs.* real distances.

These zones has been distinguished, given that when the interception of the cone is produced with a point nearer to the cone axis the measured distance is more accurate, taking into account the non-uniform sensibility of the sonar. The SRF08 is an inexpensive sonar system with a maximum range of 11 m. In order to have a more precise model of the SRF08 sensor several measurements have been carried out in a sport centre. In all, 6,500 distance measurements have been collected, taking data every 5 centimeters from a few centimeters up to three and a half meters away from a sport center wall. In the experiment the sonar has been orientated such that the ultrasonic waves intercept in a perpendicular way to the wall, avoiding echoes from other obstacles. In Figure 4, a comparison with the real data is shown. As it can be seen a good correspondence is observed between the real distances and the averages of the several measurements carried out every 5 centimeters in a straight line between the sonar and the wall. Several conclusions could be inferred from this experiment. The first conclusion establishes that the relative errors of the distance measurements are practically constant along the line between the point three and a half meters from the wall and the position of the wall. However, the absolute error increases with the distance value. In fact, this error is about 1.38 centimeters for a 3 meter distance. Because of that, in the sensor model only a maximum distance of 3 m has been considered in order to increase its accuracy. On the other hand, it is shown that distances below 10 centimeters cannot be measured. Taken into account these considerations and the sensibility zone of the sonar system indicated by the manufacturer and tested in the experiments, a cone with a half angle of 30 degrees and a maximum distance of 3 meters have been taken in the simulations. Moreover, the points of the cone which are reached first by the return wave are taken in the used sensor model. In this manner, it attempts to deal with the problem known as foreshortening. Most approaches assume the reading is along the axis of the sound wave, however, this situation occurs only if the intercepted surface is perpendicular to the sonar emitter, as in the case of the experiments of the SRF08 sonar system shown above. In the presented approach a 3D model of the cone is used, where the interceptions are taken with the nearest points in the cone. On the other side, the measurement time of the sonar is about 20 msec in the worst case. This measurement time establishes limitations, when the vehicle is in movement. That is, if the vehicle speed were 9 Km/h, a new distance would be obtained approximately every 50 mm. In order to collect the necessary data for the techniques explained in the subsequent sections, a movement of the vehicle in a straight line has been carried out. Each distance at each point is measured taking into account the cone shape of the ultrasonic sensors and the 3D geometry of the environment. Interferences with the environment are observed by a human in order to obtain the nearest distance between the sonar cone and the objects around the vehicle. In this way, a greater amount of distance data are collected for the different positions and the different sonar systems on the vehicle bodywork, in spite of the limitations pointed out above. This is a difficult task, given the human has to look for the interferences points in a 3D space and he has to assure it is the nearest interference point. Because of that, an appropriate CAD tool is essential in order to collect these data.

## 3. A Neuro-Fuzzy Approach for Environment Recognition

**Figure 5.**
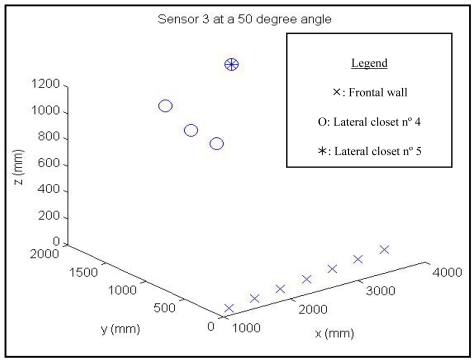
Representation of three-dimensional points corresponding to interactions between a sensor and the environment.

As it was indicated in previous section, a set of distance data has been collected. These data have been collected moving the vehicle in the 3D model shown in Section 2. Because of that, they could be seen as a spatio-temporal representation, given the data are taken while the vehicle is moving. That is, the data depend on the vehicle speed and the geometry of the environment. In the experiments it is assumed that the angular position of the sonar system is constant, although it could be changed between different trials. In Figure 5, it is shown the Cartesian coordinates of the interception points with respect to a coordinate system fixed in the vehicle. In this case, the results of the sensor numbered as 3, with an elevation angle of 50° are shown. The results for several closets and the frontal wall are presented. It could be seen that different obstacles show different patterns. Because of that, it could be devised an algorithm in order to match each pattern with each obstacle.

Note that in Figure 5 the cartesian coordinates of the interception points are presented, given that they have been extracted from the simulated environment. However, only distance values could be obtained when the real vehicle is moving. In spite of this, it seems reasonable to think that some relationship should have between the obtained distances and the detected objects, taking into account the existence of the relationship shown in Figure 5.

**Figure 6.**
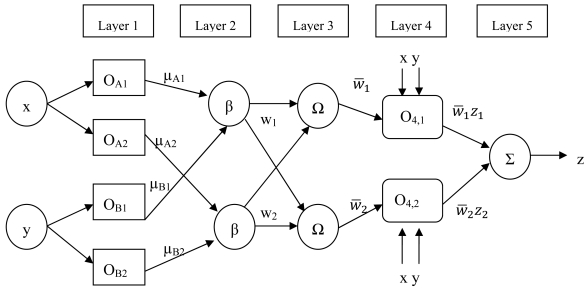
Architecture of the used adaptive neuro-fuzzy inference system.

At this point, it is necessary to look for an algorithm in order to discover these relationships. It is important to remark that the final target is an algorithm able to recognize the environment features from the relationship between the obtained distances. Hence, the chosen algorithm should recognize an environment feature with similar geometrical characteristics from the relationship between the obtained distances in other environments. In order to take common features with other environments, the attention will be focused on walls and corners features as they were defined in Section 1. Finally, a mapping between each environment feature with its own geometrical characteristics and the spatio-temporal data obtained by the 3D model will be achieved. However, it must be obtained from the spatio-temporal data and the chosen technique should have a certain degree of generalization. Because of that, some Artificial Intelligence techniques based on a training set are a good choice. One option could be the Neural Network [10,11], whose learning properties are adequate for the problem undertaken. Neural Networks have been applied successfully in many applications. In fact, their capability as universal approximators has been proven. However, the Neural Network is essentially a ‘black box’. Because of that, it has often been criticized for exhibiting a low degree of human comprehensibility. Determining exactly why it makes a particular decision is a daunting task because you have to spend time understanding the problem. Therefore, an alternative Neuro-Fuzzy approach has been chosen instead in this paper. The Neuro-Fuzzy approaches have similar learning properties as the Neural Network paradigm, in addition to, the possibility of expressing the result by rules. These are two attractive features for the problem dealt with in this paper. In addition, several researchers have also proven the good properties of Neuro-Fuzzy systems as universal approximators [11]. Different Neuro-Fuzzy approaches have been proposed in the last decades [12,13]. In this paper a Neuro-Fuzzy approach based on the scheme proposed by Jang [14] has been used. These kinds of approaches are known as Adaptive Neuro-Fuzzy Inference Systems (ANFIS systems). In this framework, a neuro-adaptive learning method has been used. That is, a given input/output data set has been used, as the first step to build a Fuzzy inference system (FIS) [15,16]. It is a method that interprets the values in the input vector based on a set of rules and assigns values to the output vector. This involves the choice of the membership functions and fuzzy logics operators, the design of fuzzy rules, the choice of the aggregation mechanism, the involvement of the fuzzy rules (inference mechanism), and finally, the defuzzification method for obtaining a numeric output. Figure 6 shows the ANFIS architecture used.

The steps to generate the initial Fuzzy Inference system are:

1. The first step is to take the inputs and determine the membership degree values to each of the appropriate fuzzy sets via membership functions. Where the input is always a crisp numerical value and the output is a fuzzy degree of membership in the interval [0,1]. In this paper, Gaussian membership functions have been used, determined by two parameters known as premise parameters.
2. The fuzzy operator is applied and a number is obtained that represents the result of the antecedent for that rule.
3. The Implication method is applied to a single number given by the antecedent, and the output is a fuzzy set represented by a membership function, which weight is a number between 0 and 1.
4. The aggregation consists of the fuzzy sets that represent the outputs of each rule are combined into a single fuzzy set. The input of this process is the list of fuzzy sets that represent the outputs of each rule, and the output is a fuzzy set.
5. The defuzzification process is applied, that is, the fuzzy set resulting from the process of aggregation, becomes as a number. In this paper, it is done by weighted average as following:
  (1) $$z=\frac{\sum_{t=1}^{N}{\bar{w}}_{i}\;z_{i}}{\sum_{t=1}^{N}{\bar{w}}_{i}}$$
  where:
  z = System output.
  z_i_= the output level of each rule
  w_i_ = the weight of each rule weighted.
  N = the total number of nodes at layer 3

The ANFIS architecture [14, 17] has been used because it is a hybrid neural network. In this case, it has five layers, where only the layers 1 and 4 are formed by adaptive nodes. That is, they have associated parameters and they can change in the training phase. In order to simplify the explanation of what happens in each layer, the structure will be particularized to the case of a system with two inputs, x and y, each one with two membership functions, μ_Ai_ y μ_Bj_, respectively, and an output z. Hence, this system is associated with two fuzzy if-then rules of Takagi-Sugeno type which are:

(2a) $$\text{If x is}\;\mu_{\text{A}1}\;\text{and y is}\;\mu_{\text{B}1}\;\text{then}\;{\text{z}}_{1}={\text{p}}_{1}\text{x}{+\text{q}}_{1}\text{y}+{\text{r}}_{1};$$

(2b) $$\text{If x is}\;\mu_{\text{A}2}\;\text{and y is}\;\mu_{\text{B}2}\;\text{then}\;{\text{z}}_{2}={\text{p}}_{2}\text{x}+{\text{q}}_{2}\text{y}+{\text{r}}_{2};$$

where *p_i_, q_i_, r_i_* are the consequent parameters.

In the first layer, each node has an output defined as:

(3) $${\text{O}}_{\text{A},\text{i}}=\mu_{\text{Ai}}(x)$$

(4) $${\text{O}}_{B,i}=\mu_{Bi}(y),i=1,\ldots ,n$$

where n is the number of membership functions of the inputs x and y, because, in this case, it is assumed that the two inputs have the same number of membership functions.

The second layer multiplies the input signals and each output of a node β corresponds to the consequent for each rule. Note that, it represents the weight of the conclusion of each rule:

(5) $$w_{i}=\mu_{Ai}(x)\mu_{Bi}(y),i=1,\ldots ,n$$

In the third layer, the output of each node Ω corresponds to the standard weights:

(6) $${\bar{w}}_{i}=\frac{w_{i}}{\sum_{t=1}^{n}w_{i}},\;i=1,\ldots n$$

The fourth layer calculates the output as a sum of the previous ones:

(7) $${\text{O}}_{4,i}={\bar{w}}_{i}z_{i}=\bar{w}(p_{i}x+q_{i}y+r_{i}),\;i=1,\ldots ,n$$

Finally, the fifth one adds all outputs of the fourth layer and it gives as output a real number:

(8) $${\text{O}}_{5,i}=\sum_{i}{\bar{w}}_{i}z_{i},\;i=1,\ldots ,n$$

In the previous paragraphs a detailed description of the ANFIS structure has been presented, however, it is necessary to specify all steps in order to get the final ANFIS system.

To sum up, it could be said that the Neuro-fuzzy modeling type ANFIS can be broken down into three main phases: collection of input/output data in a form that it will be usable by ANFIS for training, the creation of a Fuzzy System as initial structure, and the application of a learning algorithm consisting of a combination of the least-squares method and the backpropagation gradient descent method for training the ANFIS parameters. It is important to remark that these parameters are the premise and consequent parameters.

## 4. Training and Experimental Test

**Figure 7.**
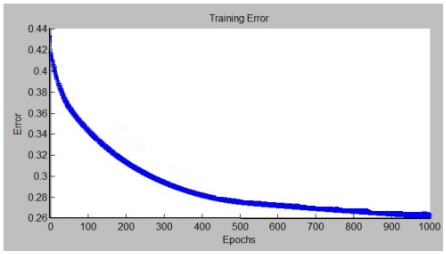
Evolution of the error curve for the training patterns.

Before developing the process described previously, the data sets have to be adapted to the ANFIS system. As it was indicated in Section 2, a spatio-temporal representation has been used as input data. In fact, the input data to the ANFIS system is the following vector (S1, S2, S3, S4, Id), where S1, S2, S3 and S4 are the four consecutive distance values and Id refers to the particular sonar system used to take these measurements. In this paper, Id has been considered as the distance in millimeters between a point on the bodywork chosen as origin and the center of the particular sonar system. Note that, each input of the ANFIS system corresponds to each component of the vector (S1, S2, S3, S4, Id). New vectors are built while the vehicle is moving. That is, with a new distance value S5 a new vector is built in this way (S2,S3,S4,S5,Id) for the same sensor. Note that, four consecutive distances values are necessary to determine the Neuro-Fuzzy output at the beginning of the path. Because of that, it is not possible the Neuro-Fuzzy to give an output in the traveled path to acquire these four first distances. Hence, a minimum secure distance should be taken in consideration at the beginning of the movement. That is, the ANFIS system is not able to provide environment features in that secure distance. In this work, a secure distance of 20 centimeters has been taken; given a vehicle speed of 9 km/h has been assumed in the simulations. In order to test the algorithms, the vehicle has been moved in a straight line towards a wall in the 3D model shown in Section 2. Taking into account the collected data, several trials have been carried out with the ANFIS system depicted in Section 3. In Figure 7 the evolution of the criterion function used in the learning phase for a trial is shown. In this case, the sum squared error between the desired outputs and Neuro-Fuzzy outputs has been considered. As it can be seen, a sum squared error of 0.26193 has been reached after 1,000 epochs.

The distance data have been divided into two groups: the training data, used in the ANFIS learning phase and the test data used to evaluate the generalization capability of the ANFIS system, after the learning phase has been finished. The training data consist of the vector (S1, S2, S3, S4, Id) and an additional component. This additional component refers to the desired output of the Neuro-Fuzzy. The desired output indicates if a particular obstacle is near the vehicle by a value in the interval [0 1].

Several trials have been carried out for different environment features, that is, a Neuro-Fuzzy system has been trained for each environment feature. In fact, wall shape and corner shape features have been treated in this paper, as a first step to build a full 3D model of the environment. At this point, it is important to precise that different wall shape features have been taken in consideration. That is, several orientations of the wall have been considered, taking one Neuro-Fuzzy system for each case. Several measurements have been carried out with the SRF08 sonar system over walls of different orientations. As it could be expected an approximate angular range of different wall orientations between zero and thirty degree allows reflecting back the ultrasonic wave towards the sonar system. Note that, the relative orientation of the sonar system is fixed with respect the vehicle and a zero wall orientation is given when the sonar system is perpendicular to the wall.

Only three wall orientations have been considered in this paper, taking into account these experimental data. It is important to remark that the target is not to determine only these three wall orientations, it is expected the Neuro-Fuzzy system is able to generalize around these orientations. Hence, the environment feature is not a wall with a fixed orientation, but it is a wall with an orientation equal or near that particular orientation. In our particular case, wall orientations angles of 4, 14 and 24 degrees were chosen. Similar considerations are applied to the corner shape environment feature.

This approach allows creating a certain number of rules in the form of Neuro-Fuzzy systems, which are able to recognize different environments features in different contexts. It is important to precise that the success of the approach depends strongly on the generalization capability of the Neuro-Fuzzy systems. That is, it is expected the Neuro-Fuzzy associated to a particular wall orientation to be able to recognize the wall in other near orientations, but it is unlikely that Neuro-Fuzzy system could be able to recognize that wall with a very different orientation. Because of that, several Neuro-Fuzzy systems are trained for several wall orientations. This approach would allow designing rules for each situation found by the vehicle in navigation. In this way, each different situation could be expressed by its own rules. This property gives more flexibility in the design process. These sets of rules along with the possibility of rotating individually the sonar systems, the determination of the environment feature distance by the sonar systems and the geometrical symmetries involved in the set of positions of the sonar systems in the vehicle could be integrated in a high-level decision system in order to support navigation tasks.

In Table 1 the results of the test data provided to the ANFIS system for determining if there is an environment feature similar to a corner are shown. This corner shape environment feature is formed by one of the closets situated on one side of the vehicle. As it can be seen satisfactory results have been achieved. It is important to point out that only data provided from the simulated environment have been used. D refers to the distances of the vehicle from the origin point of its path and (S1, S2, S3, S4, Id) are the inputs to the Neuro-Fuzzy system, where S1,S2,S3 and S4 are the consecutive readings of the sonar sensor and Id refers to the used sonar system. Id is expressed as a distance in millimeters between a point taken as origin on the vehicle bodywork and the centre of that particular sonar system. In the following, the term geometrical features will be used to refer to a particular *a priori* unknown relationship between the consecutive readings included in a particular input vector.

Therefore, the column named as “estimated output” refers to the level of success about the detection of the geometrical features for a particular environment feature. Note that, if the level of success is high, it is very likely that particular environment feature exists in the neighborhood of the vehicle. It is necessary to give these output values in the learning process and they are introduced by the expert for each Neuro-Fuzzy system, who determines if all received values correspond with the trained element or not. As it was pointed out above, the Neuro-Fuzzy output indicates if the geometrical features corresponding to a particular environment feature are found in the neighborhood of the vehicle. Since each Neuro-Fuzzy system is trained to detect the geometrical features of a different environment feature. A value of “one” in a particular Neuro-Fuzzy output points out that the geometrical features of this environment feature are similar to the geometrical features corresponding to the environment feature associated with this particular Neuro-Fuzzy system in the neighborhood of the vehicle. On the contrary, a value of “zero” indicates that the geometrical features of the corresponding environment feature have not been detected. So, the outputs with intermediate values mean that some of the readings of the sonar sensor refer to this environment feature, and the other readings belong to an unknown environment feature for this Neuro-Fuzzy system.

It is important to consider the fact that each input vector is a spatio-temporal representation. It is possible some readings of the sonar sensors detect the environment feature and others detect a different environment feature. Because of that, the estimated output is not only taken as one or zero, rather than the ratio between the number of sonar sensor readings corresponding to a positive detection and the total number of readings, in our case four for each sensor. For example, the estimated output of 0.5 shown in Table 1 means that the corner is being detected by the first two readings and the other two ones correspond to another element. Because of that, the ratio is calculated as the division between 2 and the total number of readings, four in our case. However, the expert sometimes introduces a different value than the resultant value of applying the method depicted before. For example, the estimated value 0.15 pointed out in Table 2 match with the situation where only the wall is detected by S4. If the method depicted above were applied the result of the division would be 0.25. However, the expert realizes that situation in comparison with other similar ones is less clear. Because of that, a value of 0.15 has been assigned instead of 0.25. In Table 3 a more pictorical description is presented. In this case, three equivalent situations are shown where a wall with an orientation of zero degree is being detected, after the vehicle has passed near a lateral wall. The results collected in the first row correspond to when the vehicle is passing a lateral wall and the ultrasonic waves of this particular sonar system are always being reflected by the lateral wall. Because of that, the estimated output is zero and the distance S1, S2, S3 and S4 are taking the same values. In the case of the second row, the distances S1, S2 and S3 points out a lateral wall, while, S4 is a different distance pointing out a wall with a zero orientation is present. If the methods depicted above were followed an estimated value of 0.25 should be taken. However, it is important to observe the difference between S4 and the previous value S3 in the sense that a greater difference indicates the new value really corresponds to a wall at zero degree orientation and it is not a result of the noise in the measurements. Because of that, a value of 0.15 is chosen instead of 0.25. Similar arguments are applied in the results shown in the third row, where S3 and S4 are different from S1 and S2. In this case a value of 0.5 for the estimated value would be obtained applying the method explained above. However, the small different of 17 mm between S2 and S3 advises to choose a lower value. Because of that a value of 0.3 has been chosen. The particular election depends on the expert perception. This way of dealing with the trained data allows including subjective perceptions of the experts such that the final system takes decisions in a more intelligent way from the human point of view. Additionally, it is important to point out that the Neuro-Fuzzy output data shown in table 1 correspond to the Neuro-Fuzzy system, after the training phase has been finished. That is, a comparison between the expected values, extracted from the 3D model and the Neuro-Fuzzy outputs is shown in Table 1. In Table 2 similar results are shown for determining a wall in a specific orientation.

**Figure 8.**
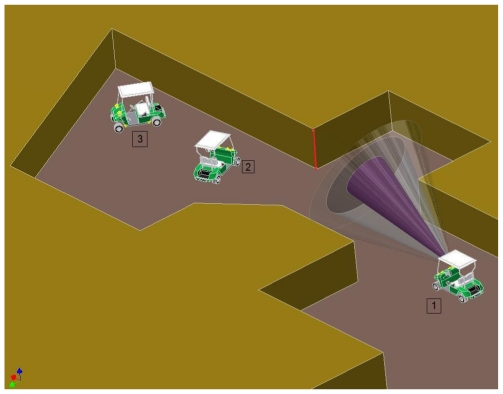
Image of the new scenario.

It is important to point out that Tables 1 and 2 show the outputs of the Neuro-Fuzzy system for input values never seen by the Neuro-Fuzzy system. That is, these values have not used in the training phase. Hence, the shown data could be considered as a way of determining the generalization properties of the Neuro-Fuzzy system. Because of that, some outputs differ in a certain amount of the expected values. It is the case pointed pot in Table 1, where the output is 0.4652 and the expected value is 1. However, only a difference around one of ten is found in most of the other cases. In order to prove the methods in other simulated context, a different scenario has been designed as it is shown in Figure 8. New situations are found in this new scenario. Several Neuro-Fuzzy systems for each different environment feature are pointed out by a number in Figure 8.

The results of the ANFIS system for a corner shape environment feature marked in the Figure 8 with a red line are shown in Table 4. This Neuro-Fuzzy system is indicated as 1 in Figure 8. The shown results in the column labeled as “Neuro-Fuzzy output” corresponds to the vehicle following a path in a straight line, as it is shown in the Figure. As it can be seen, the Neuro-Fuzzy outputs take a value near one when the vehicle is near the environment feature and these values decrease when the vehicle is passing the corner.

As it can also be seen, the format of Table 4 is different than the previous ones shown in Tables 1 and 2. In this case, there is not an estimated output column. In the previous cases, the estimated output data was extracted from the simulated scenario, given most of them were used in the training phase. In this case, a new scenario is presented to the Neuro-Fuzzy system in order to evaluate the generalization capability in a different scenario than the scenario used for training. Because of that, this column has been removed, given it could be directly inferred from the Figure 8. Note that, S1, S2, S3 and S4 are distances in millimeters from the position where the vehicle is drawn up to the corner shape environment feature pointed out with a red line, while Id indicates the distance of the used sonar system in millimeters to an origin on the vehicle bodywork. Note that, the origin was chosen as the center of the sonar system, numbered as 7.

**Figure 9.**
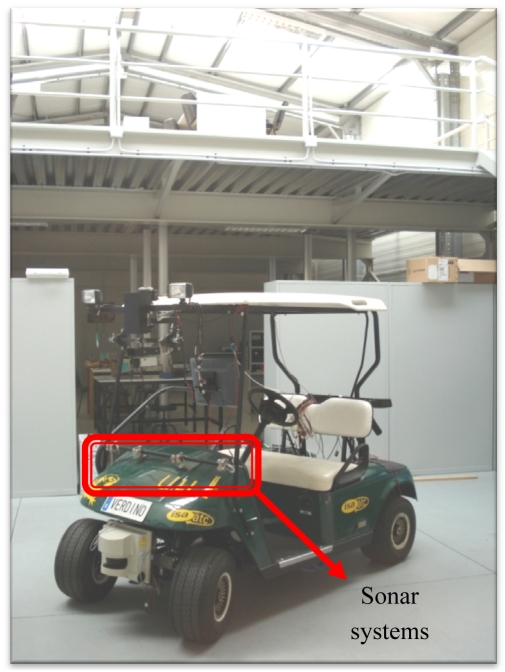
Photograph of the storework, the vehicle and the sonar systems.

In order to prove the robustness of the methods, several data were acquired in a real environment. In Figure 9 a photograph of the vehicle, the sonar systems and the real environment is presented. As it can be seen in Figure 9, a passenger shuttle manufactured by EZGO has been used in the experiments. Particularly, eight ultrasonic devices controlled by a microprocessor AT90S8515 have been used to acquire the sonar data and these data are sent to a PC system in order to carry out the higher level processing. In fact, the Neuro-Fuzzy systems have been implemented by the software in the PC system.

In the real case, several effects negatively influence the obtained distances. In fact, specular reflections could appear and the surface texture influences over the measurements. In the sensor model used in the simulations these specular reflections have not been considered, hence, some erroneous readings are obtained overall in highly reverberant environments. The sensor model has been elaborated based on the data obtained from the experiments depicted in previous paragraphs. That is, a low reverberation environment has been used. The specular reflections are not taken into account in the 3D model of the sonar system. This is a drawback in order to implement this approach in a real environment, where there are specular reflections. However, it is important to remark that specular reflections are linked to a particular scenario, that is, it is very likely different specular reflections are produced in different scenarios. Because of that, the used 3D Model of the sonar system does not include specular reflections in order to increase the generalization capability of the Neuro-Fuzzy system in other scenarios. Hence, it is necessary to remove the specular reflections in this case. Instead of taking the data in a raw way, a previous processing is carried out over the collected readings. Taking into account that short range data are more reliable than long range data in the case of ultrasonic sonars and the specular reflections attempts to give longer distances, the following process has been carried out: the distances between the different readings have been calculated, including the corrections given the vehicle is moving. After that, only the values below a certain threshold have been taken in order to obtain the average over the four consecutive distances values. Furthermore, a weighted average has been used, where the shorter distances have been associated with higher weights in the weighted average. It is important to remark that the readings are obtained from different positions, given the vehicle is moving. This fact makes this technique convenient in order to eliminate specular reflections.

**Figure 10.**
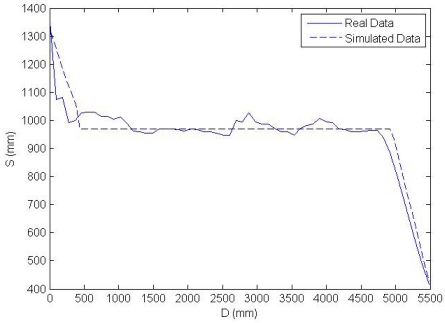
Distances measured by one of the sonar systems *vs.* vehicle position. The Average of distances has been used for the real sonar data and the raw distances has been used for the data extracted from the simulated environment.

Taking into account the previous considerations, it is necessary to have seven real distance values to get the Neuro-Fuzzy input vector (S1, S2, S3, S4, Id) at the beginning of the movement. However, after that, it is only necessary to have one additional real reading each time, given the new average is obtained taking the previous readings. In this way, a new Neuro-Fuzzy output value is available approximately each 20 milliseconds in the worst case. In Figure 10 a comparison of the average values and the data extracted from the simulated environment is presented. As it can be seen in the graph, roughly, three zones could be distinguished. The first curve refers to the corner formed by the closet at the middle of the path, as is shown in Figure 9. While the second line corresponds to the lateral closets after the corner. Finally, the last steep curve appears when a frontal wall is being detected.

In Table 5, the results of the trained ANFIS system for determining the wall when the average of the real data is used as inputs are shown. The values of the Neuro-Fuzzy output for these real data, taken as inputs, could be seen in the column named “Test outputs”. In this case, the estimated outputs have been calculated as it was pointed out above using the equivalent situation in the simulated environment. As it can be seen several differences are appreciated between the estimated outputs and the test outputs given specular reflections and surface texture dependency appears in the real case. Nevertheless, a correspondence can be established with the degree of wall detection in most cases, although a method has been considered to eliminate most of the specular reflections. In addition to there is an additional real problem when some previous echoes of a previous reading reaches the sonar receiver at the current reading. In this case, a shorter distance than the real one is obtained. This troublesome effect could be overcome by increasing the measurement time of the sonar. However, this increase will introduce limitations in the approach; given a smaller amount of data will be collected. In order to overcome this problem the Neuro-Fuzzy system should be trained with real data in the case of environment features with so many reflections at close ranges.

In Table 6, the results of the trained ANFIS system for determining a corner when the average of the real data is used as inputs are also shown. Similar observations can be done in this case. On the other side, it is important to remark that different vehicle speeds than the ones assumed in the simulations have been used in the experiments. However, these speeds are near to 9 Km/h, given this speed was chosen in the training phase. Because of that, it is expected the values of the Neuro-Fuzzy system outputs differs in both cases. In spite of these difference assumptions over the vehicle speed a correspondence between the estimated outputs and the test outputs is observed. It reveals the generalization properties of the Neuro-Fuzzy approach in this case. In order to test the generalization of the Neuro-Fuzzy system a different real scenario has been taken.

**Figure 11.**
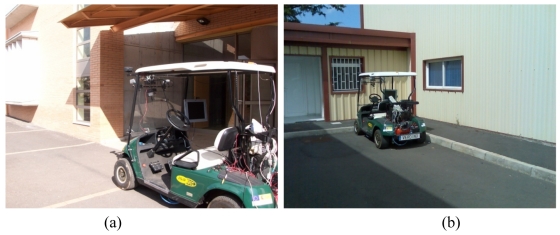
Photographs of the other scenarios used in the experiments: (a) building entrance (b) frontal wall with a lateral wall.

As it can be seen in Figure 11a the entrance of a building has been chosen as a real scenario. In this case, the Neuro-Fuzzy system to detect a corner shape environment feature and the Neuro-Fuzzy system to detect a wall have been used. As it can be seen in Figure 11b, these two environment features can be distinguished in the entrance. In Table 7 it could be seen that the Neuro-Fuzzy system for the corner shape environment feature give values greater than one at the beginning of the path. This fact, it could be interpreted as the environment feature is not being recognized given the output values are greater that one, the limit of the output training data. However, when the vehicle goes forward the environment feature is detected and when the vehicle passes the environment feature these values decrease.

Note that, at the end a negative value is obtained, indicating the Neuro-Fuzzy systems do not find the corner shape environment feature. In the case of the Neuro-Fuzzy for the wall environment feature, the wall is detected at the beginning, after that, the output values are smaller than one or they are out of the interval [0 1], indicating that the environment feature is not being detected in the neighborhood of the vehicle. In Figure 11b another real scenario including a frontal wall is shown. As it can be appreciated in Table 8, the outputs values of the Neuro-Fuzzy system are increasing when the vehicle is going towards the wall. Note that, the estimated output column has been removed as in Table 3. Moreover, in order to evaluate the exact estimated values a 3D model should be built for each case. Because of that, the expected values are inferred from the observation of this particular scenario by the expert and the relative position of the vehicle in the environment. At this point, it is important to precise that the scenarios chosen in Figure 11a,b are different scenarios than the scenario used in the training phase from the geometrical point of view. However, similar environment features are presented in that environment. On the contrary, if there were not any common environment feature with the training scenario, the Neuro-Fuzzy system would be unable to detect those new environment features, given they have not been included in the training, at least in any way.

## 5. Conclusions

In this paper, an approach based on a simulated environment and the application of a Neuro-Fuzzy technique in order to extract features of the environment by ultrasonic sensors has been presented. A three dimensional model of the ultrasonic cones has been considered. An experiment has been carried out in order to determine the characteristics of the SRF08 sonar system in more detail. Several trials have been done taking into account simulated data and several experiments have been done using real data. Satisfactory results have been achieved in the simulated case. Furthermore, real experiments taking other speed conditions and other environments have been carried out. These experiments have proved the good generalization properties of the used approach. However, it is important to remark a constraint in the application of the proposed method. It is that the vehicle has to wander at low speed in order to collect a sufficient number of data, although spatio-temporal vectors have been used in this work. On the other hand, although several zones has been distinguished in the cone formed by the ultrasonic wave at the first stage of the work, only interceptions with the exterior zones have been observed in the experiments. In order to observe interceptions with other zones the angle of the sonar system has to change and the resultant interceptions are produced on the vehicle bodywork. Because of that, an accuracy index based on the interception zone has not been included in this work. Finally, the approach has been useful to detect and extract features of the big environment features, whereas the feature extraction performance of other smaller environment features such as cans is highly limited by the wide width of the sonar cone. In spite of these drawbacks, the trained Neuro-Fuzzy systems could be integrated in a high-level decision system as a set of rules in order to support navigation tasks. This is usual in behavior-based approaches in robotics. Nevertheless, the techniques presented in this paper must be seen as a complementary tool in order to explore the environment, supporting other environment recognition approaches.

## Figures and Tables

**Table 1.** Outputs of the Neuro-Fuzzy system for test patterns in the case, where a corner is being detected. S1, S2, S3, S4, Id and D are given in millimeters.

**D**	**S1**	**S2**	**S3**	**S4**	**Id**	**Estimated output**	**Neuro-Fuzzy output**
504	4,298	4,258	4,170	4,170	880	0.6	0.5645
504	2,241	3,778	3,717	3,657	0	0.8	0.7504
1,008	3,756	3,721	3,693	3,693	660	0.5	0.4826
1,323	3,165	3,112	3,060	3,009	220	1	1.0903
2,205	2,321	2,282	2,251	2,235	0	1	0.4652
3,339	4,170	4,170	4,170	4,170	880	0	0.0050
5,922	2,277	2,214	2,151	2,088	660	0	0.0121
6,489	1,710	1,647	1,584	1,521	0	0	0.0056

**Table 2.** Outputs of the Neuro-Fuzzy system for test patterns in the case, where a wall is being detected. S1, S2, S3, S4, Id and D are given in millimeters.

**D**	**S1**	**S2**	**S3**	**S4**	**Id**	**Estimated output**	**Neuro-Fuzzy output**
1,071	4,170	4,170	4,170	4,170	880	0	-0.0013589
1,827	3,693	3,693	3,693	3,693	660	0	-0.0064743
2,583	2,727	2,727	2,727	2,727	220	0	0.00056447
4,536	3,663	3,600	3,537	3,474	660	0.6	0.8715
5,292	2,727	2,727	2,727	2,710	220	0.15	0.042024
5,796	2,403	2,340	2,277	2,214	880	1	1.059
5,985	2,214	2,151	2,088	2,025	0	0.8	0.91389
6,363	1,828	1,765	1,702	1,639	220	1	1.0048

**Table 3.** Setting the estimate outputs where the transition between a lateral wall and a wall with zero degree orientation is taking place. S1, S2, S3, S4 and Id are given in millimeters.

**Id**	**S1**	**S2**	**S3**	**S4**	**Estimated Output**
220	2,727	2,727	2,727	2,727	0
220	2,727	2,727	2,727	2,710	0.15
220	2,727	2,727	2,710	2,647	0.3

**Table 4.** Outputs of the Neuro-Fuzzy system for test patterns in the case, where a corner shape environment feature is being detected in a new scenario.

**S1**	**S2**	**S3**	**S4**	**Id**	**Neuro-Fuzzy output**
6752	6664	6577	6490	0	0.7655
6664	6577	6490	6404	0	0.7383
6577	6490	6404	6320	0	0.7004
6490	6404	6320	6236	0	0.6653
6404	6320	6236	6153	0	0.6348
6320	6236	6153	6072	0	0.5904
6236	6153	6072	5992	0	0.5428

**Table 5.** Outputs of the Neuro-Fuzzy system for real data and the simulated data in the case, where a wall is being detected. S1, S2, S3, S4, Id and D values are given in millimeters.

**D**	**S1**	**S2**	**S3**	**S4**	**Id**	**Estimated output**	**Test output**
2,623.32	917.33	906.07	2,667.5	805.07	220	0	-1.2
4,566.52	1,123.82	1,113.25	1,074.97	1,055.74	660	0.6	1.004
5,732.4	528.2	451.1	383.5	349.1	880	1	0.687
6,023.92	677.05	544.18	524.95	4,49.95	0	0.8	0.522
6,315.4	493.07	433.13	357.95	226.11	220	1	0.719

**Table 6.** Outputs of the Neuro-Fuzzy system for real data and the simulated data in the case, where a corner is being detected. S1, S2, S3, S4, Id and D values are given in millimeters.

**D**	**S1**	**S2**	**S3**	**S4**	**Id**	**Estimated output**	**Test output**
582.96	1,065.8	1,055.1	1,324.6	1,686	880	0.6	0.634
1,068.76	999.96	971.20	1,092.47	1,614.08	660	0.5	0.25
1,360.24	3,412.6	892.21	3,416.6	3,414.5	220	1	0.70
3,400.6	801	1,110.8	1,020.1	1,059.6	880	0	-0.39
5,926.76	832.26	739.58	664.40	581.07	660	0	-0.22

**Table 7.** Outputs of the Neuro-Fuzzy systems for real data when the vehicle is passing the entrance of a building as it is seen in Figure 11a.

**S1**	**S2**	**S3**	**S4**	**Id**	**Neuro-Fuzzy for corner shape environment feature**	**Neuro-Fuzzy for wall environment feature**
2,370.1	2,187.7	1,995.2	1,818.7	220	5.148	1.1051
2,187.7	1,995.2	1,818.7	1,628.5	220	4.6825	0.9418
1,995.2	1,818.7	1,628.5	1,453.3	220	3.1802	0.95318
1,818.7	1,628.5	1,453.3	1,453.3	220	1.0401	0.57518
1,628.5	1,453.3	1,453.3	1,148.3	220	1.2071	1.2076
1,453.3	1,453.3	1,148.3	988.53	220	0.92475	0.61625
1,453.3	1,148.3	988.53	845.26	220	0.81248	0.74335
1,148.3	988.53	845.26	751.71	220	0.21002	1.7613
988.53	845.26	751.71	1,883.1	220	-4.1442	-1.7194

**Table 8.** Outputs of the Neuro-Fuzzy systems for real data when the vehicle is going towards a frontal wall as it is seen in Figure 11b.

**S1**	**S2**	**S3**	**S4**	**Id**	**Neuro-Fuzzy for wall environment feature**
549.55	668.21	601	638.94	0	0.4687
668.21	601	638.94	637.9	0	0.5081
601	638.94	637.9	654.7	0	0.5545
638.94	637.9	654.7	630.45	0	0.5813
637.9	654.7	630.45	697.14	0	0.5243
654.7	630.45	697.14	707.88	0	0.6851
630.45	697.14	707.88	635.47	0	0.7460
697.14	707.88	635.47	722.44	0	0.5334
707.88	635.47	622.44	631.1	0	0.7136
635.47	622.44	631.1	579.3	0	0.8089

## References

[1] Borrmann D., Elseberg J., Lingemann K., Nüchter A., Hertzberg J. (2008). Globally consistent 3D mapping with scan matching. J. Robot. Auton. Syst..

[2] Song K., Chen C., Huang C.C. Study of an ultrasonic sensor system for lateral collision avoidance at low speeds.

[3] Tardos J.D., Neira J., Newman P., Leonard J. (2002). Robust mapping and localization in indoor environments using sonar data. Int. J. Robot. Res..

[4] Ribas D., Neira J., Ridao P., Tardos J.D. SLAM using an imaging sonar for partially structured environments.

[5] Wijk O., Christensen H.I. (2000). Localization and navigation of a mobile robot using natural point landmarks extracted from sonar data. J. Robot. Auton. Syst..

[6] Barshan B. (2008). Objective error criterion for evaluation of mapping accuracy based on sensor time-of-flight measurements. Sensors.

[7] Auran P.G, Silven O. (1996). Underwater sonar range sensing and 3d image formation. Control Eng. Practice.

[8] Jaroš J., Rozman J. (2004). Three-dimensional measured ultrasound field. Meas. Sci. Rev..

[9] Autodesk® Inventor™.

[10] Pajares G. (2006). A Hopfield Neural Network for Image Change Detection. IEEE Trans. Neural Netw..

[11] Ying H. General Takagi-Sugeno fuzzy systems are universal approximators.

[12] Mitra S, Hayashi S. (2000). Neuro-fuzzy rule generation: Survey in soft computing framework. IEEE Trans. Neural Netw..

[13] Marichal G.N., Acosta L., Moreno L., Mendez J.A., Rodrigo J.J., Sigut M. (2001). Obstacle avoidance for a mobile robot: a neuro fuzzy approach. Fuzzy Sets Syst..

[14] Jang J.R. (1993). ANFIS: Adaptive-network-based fuzzy inference system. IEEE Trans. Syst. Man Cybern..

[15] Zadeh L.A. (1989). Knowledge representation in fuzzy logic. IEEE Trans. Knowl. Data Engin..

[16] Guijarro M., Pajares G. (2009). On combining classifiers through a fuzzy Multicriteria Decision Making Approach: applied to natural textured images. Expert Syst. Appl..

[17] MathWorks Matlab. http://www.mathworks.com/products/fuzzylogic/.

[18] Wall R.W., Bennett J., Eis G., Lichy K., Owings E. Creating a low-cost autonomous vehicle.

[19] Silver D., Morales D., Rekleitis I., Lisien B., Choset H. (2004). Arc carving: obtaining accurate, low latency maps from ultrasonic range sensors.

[20] Hori T., Nishida Y. Improvement of position estimation of the ultrasonic 3D tag system.

[21] Rencken W.D. Concurrent localisation and map building for mobile robots using ultrasonic sensors.

[22] Hong M.L, Kleeman L. (1997). Ultrasonic classification and location of 3D room features using maximum likelihood estimation—Part II. Robotica..

[23] Kleeman L. (1989). Ultrasonic automonous robot localisation system.

[24] Barshan B. (2007). Directional processing of ultrasonic arc maps and its comparison with existing techniques. Int. J. Robot. Res..

[25] Elfes A. (1987). Sonar based real-world mapping and navigation. IEEE T. Robotic. Autom..

[26] Wijk O., Christensen H.I. (2000). Triangulation-based fusion of sonar data with application in robot pose tracking. IEEE T. Robotic. Autom..

[27] Leonard J.J., Durrant-Whyte H.F. (1992). Directed Sonar Sensing for Mobile Robot Navigation.

[28] Kuc R., Siegel M.W. (1987). Physically-based simulation model for acoustic sensor robot navigation. IEEE T. Pattern Anal..

[29] Baskent D., Barshan B. (1999). Surface profile determination from multiple sonar data using morphological processing. Int. J. Robot. Res..

[30] Altun K., Barshan B. (2008). Performance evaluation of ultrasonic arc map processing techniques by active snake contours. Springer Tracts in Advanced Robotics (STAR) Series.

